# Engaging diverse patients in a diverse world: the development and preliminary evaluation of educational modules to support diversity in patient engagement research

**DOI:** 10.1186/s40900-023-00455-0

**Published:** 2023-07-07

**Authors:** Erin E. Michalak, Iva W. Cheung, Elsy Willis, Rachelle Hole, Beverley Pomeroy, Emma Morton, Sahil S. Kanani, Steven J. Barnes

**Affiliations:** 1 Department of Psychiatry, University of British Columbia, 420-5950 University Boulevard, Vancouver, BC V6T 1Z3 Canada; 2 School of Social Work, University of British Columbia Okanagan, Kelowna, BC Canada; 3 Fraser Health Authority, Mental Health and Substance Use, Surrey, BC Canada; 4 Department of Psychology, University of British Columbia, Vancouver, BC Canada

**Keywords:** Educational modules, Patient-oriented research, Methods, Diversity, Inclusion, Hardly reached

## Abstract

**Background:**

Current practices for engaging patients in patient-oriented research (POR) result in a narrow pool of patient perspectives being reflected in POR. This project aims to address gaps in methodological knowledge to foster diversity in POR, through the co-design and evaluation of a series of educational modules for health researchers in British Columbia, Canada.

**Methods:**

Modules were co-created by a team of academic researchers and patient partners from *hardly*-reached communities. The modules are presented using the Tapestry Tool, an interactive, online educational platform. Our evaluation framework focused on engagement, content quality, and predicted behavior change. The User Engagement Scale short form (UES-SF) measured participants’ level of engagement with the modules. Survey evaluation items assessed the content within the modules and participants' perceptions of how the modules will impact their behavior. Evaluation items modeled on the theory of planned behavior, administered before and after viewing the modules, assessed the impact of the modules on participants’ perceptions of diversity in POR.

**Results:**

Seventy-four health researchers evaluated the modules. Researchers’ engagement and ratings of module content were high. Subjective behavioral control over fostering diversity in POR increased significantly after viewing the modules.

**Conclusions:**

Our results suggest the modules may be an engaging way to provide health researchers with tools and knowledge to increase diversity in health research. Future studies are needed to investigate best practices for engaging with communities not represented in this pilot project, such as children and youth, Indigenous Peoples, and Black communities. While educational interventions represent one route to increasing diversity in POR, individual efforts must occur in tandem with high-level changes that address systemic barriers to engagement.

**Supplementary Information:**

The online version contains supplementary material available at 10.1186/s40900-023-00455-0.

## Plain Language summary

Patient-Oriented research (POR) recognizes patients as partners in the research process, contributing valuable knowledge and lived experience to improve health outcomes and research translation. Unfortunately, POR presently does not represent all patients: patient partners involved in research tend to be white, middle-class women. Limited diversity in POR may limit research impact, in part because health research is not fully inclusive of the people requiring healthcare services and support. Although they recognize the need for diversity, many health researchers do not understand how to foster this. To empower health researchers with tools and knowledge to foster diversity in POR, we coordinated teams that co-created a set of educational modules. These teams included academic researchers and patient partners from seldom-heard communities in health research. The modules were built using the Tapestry Tool, an interactive, educational platform. We evaluated the modules using online surveys of 74 health researchers for three things: quality of module content, how engaging they were, and how they impacted health researchers’ views on diversity in POR. Our results show that the modules' content was high quality and engaging. After viewing the modules, health researchers were more likely to feel like they had a role in engaging with more diverse people in their research, and felt that they had the tools, knowledge, and ability to do so. Education and knowledge for health researchers are a first step to increasing diversity in POR settings, but systemic barriers to inclusion also need attention.

**Supplementary Information:**

The online version contains supplementary material available at 10.1186/s40900-023-00455-0.

## Background

Patient-Oriented Research (POR) aims to improve health outcomes and research translation through collaboration with patients as research partners [1]. The Canadian Strategy for Patient-Oriented Research (SPOR) defines POR as “meaningful and active collaboration in governance, priority setting, conducting research and knowledge translation,” with patient partners being defined as “individuals with personal experience of a health issue and informal caregivers, including family and friends [1].” POR offers one potential solution to the low implementation rates of health research, with estimates that up to 85% of health research may be wasted [2]. Additional potential benefits of POR may include improved relevance of research to patients, increasing accessibility of study designs, improved informed consent processes, increased rates of enrollment and retention, increased public trust in health research, and benefits to patient partners through fostering self-esteem and a sense of identity [3–7].

Unfortunately, POR does not currently represent all patients: patient partners commonly involved in POR do not reflect the diversity of the population the research aims to serve [8]. This limited diversity limits research impact, partly because health research does not adequately respond to the needs of the people accessing healthcare services and support. While many health researchers recognize the need for diversity in patient engagement, many do not understand how to foster this diversity in practice. This is compounded by a paucity of resources specific to engaging different historically marginalized communities in health research. This project sought to provide health researchers tools and knowledge to help them support diversity in POR through actionable and sustainable ways. Honouring the importance of the patient voice, these modules have been co-created by blended teams of patient partners, academic researchers, and research trainees from diverse communities.

### Positioning our understanding of diversity in this project

In the context of this project, we acknowledge that fostering diversity intersects closely with inclusion. Our definition of diversity is thus more holistic and is adapted from the definitions of diversity and inclusion from The Canadian Centre for Diversity and Inclusion [9]. In this project, we understand diversity as advancing the creation of spaces and relationships that foster, embrace, respect, and accept a variety of dimensions, qualities and characteristics that make up the individual. These include, but are not limited to, race, ethnicity, age, gender, sexual orientation, religious beliefs economic status, physical abilities, life experiences, and other perspectives reflected in individual diversity.

### Diversity in POR: where we are now

A foundational step in patient engagement is the recruitment of patients as research partners. Common recruitment practices include social media and health systems outreach, which tend to recruit a narrow pool of patient partners: retirement-age, middle-class, white women [10–12]. These patient partner demographics are unrepresentative of the broader population: in British Columbia, Canada, for example, 30% are immigrants, 30% speak a language other than English or French, 21% of the population between 20 and 65 years of age live with a disability, and 14% live rurally [13, 14]. Individuals and communities who face the most barriers to engaging in health research may include those who are racialized, are disabled, are LGBTQ2S+ , are homeless, have low health literacy, and use communication aids [15]. Fergusson et al. [16], in their review of the application of patient engagement in published clinical trials, report that 26% of studies included engagement of racialized patients on the research team, and 48% of studies included racialized populations in their study. However, this does not directly translate into representative engagement or guarantee that power imbalances and tokenism were addressed [16]. While some studies describe specific methods for engaging with racialized communities, methods tend to be limited to simple strategies like having meetings outside of office hours or visiting the target community [16]. Even when patients from equity-deserving communities are included in POR, they may not be engaged meaningfully or authentically, possibly because of a lack of resources or understanding, structural power imbalances, or exclusionary engagement practices [2, 10]. Sayani et al. [10] and Golenya et al. [17] offer methods for improving diversity in patient and public involvement research. However, while valuable, suggested recruitment strategies are limited to using clear, jargon-free recruitment materials, and distributing recruitment flyers through various channels.

In addition to a lack of diversity among patient partners in POR, lack of diversity among research team members can make it difficult to foster the inclusion of diverse communities in POR. For example, Johnston et al. [18] reference that a lack of representation of diverse faculty members in Canadian universities reduces the likelihood that people with lived experience will participate in laboratory-based POR. This is attributed to increased discomfort and disconnect from the research team during participation, and the possibility of not feeling heard or valued during the research process. The issue of intersectionality is also important when considering discrimination on the basis of gender and race, especially considering the overrepresentation of white, middle-class women in health research and advocacy work compared to women of colour. Kumanyika et al. [19] suggest that minority women do not share the same privileges as white women, including an understanding of the dominant culture, as well as sharing the obligations associated with participating in it. When considering other intersecting identities including socioeconomic status, disabilities and sexual identity, among others, the inclusion of diverse patient partners in health research calls for a parallel effort in fostering the inclusion of diverse research team members in POR as well.

The lack of diversity within POR has consequences for the applicability of research, including preventing researchers from fully encapsulating the perspectives and experiences of the patient population. This limits the ability to generalize results beyond communities involved in the research process, and reinforces inequities in healthcare systems [10, 17, 20]. Including a narrow pool of patient partners, as well as research team members, in health research may further the lack of mentorship, trust, and capacity building for members of *hardly*-reached communities, thereby perpetuating the lack of diversity in health research [21]. Furthermore, teams with diverse members, including patient partners, may be better equipped to address health disparities among patients and may have members with lived experience of the highest burden of disease [11, 22]. Case studies provided by the Canadian Foundation for Healthcare Improvement (2019) suggest that engaging diverse groups of patient and community partners can help to build and strengthen relationships, foster trust, encourage self-awareness, and cultivate acceptance during the processes of healthcare improvement and systems change. Thomson et al. [23] argue that excluding diverse patients from research might result in a “more paternalistic application of guidelines” to such groups, thereby reinforcing existing health inequities. Patients and practitioners from similar social and educational backgrounds are more likely to have shared values, facilitating shared decision making and disregarding values that may be different from theirs. This reinforces inequities two-fold: by excluding certain groups from decision making in research, and by ignoring the needs, wants, and values of groups not part of the dominant culture. Taken together, these barriers are significant and contradict the goals of POR: to improve the implementation of health research within the healthcare system, thereby improving health outcomes and decreasing health inequities.

### Current research gaps in effective patient engagement

Despite increased emphasis on the importance of POR, there remain important research gaps in terms of benefits, limitations, and best practices for effective patient engagement [6, 16]. Health researchers may be hesitant to engage with patients, due to beliefs that patient engagement results in unfeasible changes to research scopes and increased time and cost, or challenges such as unclear patient partner roles, and a lack of clear conflict-resolution practices [2–4, 6, 16]. More evidence-informed resources that provide researchers with tools and knowledge to engage patients, successfully recruit and retain patient partners, or decide what type of engagement methods are most appropriate for a given project are needed [6, 12, 16]. These resources are further limited regarding specific methods of engaging with historically marginalized communities. Best practices in POR require development due to a lack of comparative analysis between various methods [6, 16]. Furthermore, reporting of methodology and rationale in studies that include patient engagement can be sub-optimal [16]. The use of traditional research practices may perpetuate the lack of diversity in POR: many equity-deserving communities are *hardly*-reached through traditional recruitment methods such as convenience or snowball sampling [24]. Limited reporting of methods in POR leaves knowledge users with little understanding of how the research is conducted, thereby decreasing the reproducibility, applicability, and impact of POR [16].

The objective of this project was to develop and evaluate a series of online educational modules designed to support health researchers engaging with diverse people in POR, in collaboration with patient partners. The modules focus on providing important context and practical tools for engaging with diverse communities across health research settings.

## Methods

This project was funded by the Patient Engagement Methods Cluster of the British Columbia Support for People & Patient-Oriented Research & Trials Unit (BC SUPPORT Unit) in Canada. The BC SUPPORT Unit supports, streamlines, and increases POR in British Columbia, as part of a national Strategy for Patient-Oriented Research (SPOR) led by the Canadian Institutes of Health Research (CIHR). For more information on how impact of patient engagement was reported in this study, see the Guidance for Reporting Involvement of Patients and the Public (GRIPP2) Short-Form checklist [25] in Additional file 1.

### The Tapestry Tool platform

We created a suite of educational modules hosted on the Tapestry Tool platform [26], developed by co-author SJB. The Tapestry Tool differs from other educational resources in that it is adaptable to individual needs and is bidirectional. The platform allows for multiple types of content to be displayed, including HTML5 Package (H5P) interactive content [27], videos, and PDFs. Learners can rearrange the order of topics according to individual preference or learning style, and contribute their own content. This bidirectional nature of the Tapestry Tool allows the content to be continually improved and updated. Finally, creating an online resource is cost-effective for creators and convenient for learners, who can view materials at any time with an electronic device.

### Module topic identification

Iterative methods were used to identify and prioritize module focus areas. First, the core team (authors EEM, IWC, RH and patient author BP; see [28] for further information on how the team members positioned themselves in this project) worked collaboratively to identify potential module focus areas; an assumption of the team was that there would be value in taking a semi-sequential approach to module development and that the knowledge gained from developing the first modules would help inform the development of subsequent modules. Two modules were selected for initial development: a “primer” highlighting the value of fostering diversity in POR and a module focused on members of LGBTQ2S+ communities. Second, an environmental scan was conducted (August to September 2019, authors EM and IWC) with the goal of identifying where marked gaps exist in resources designed to support diversity in POR. This scan included groups not represented in the final module topics, including young people, people who use illicit substances, and Indigenous populations. Third, a series of consultations were conducted with BC SUPPORT Unit stakeholders (e.g., patient partners, health researchers, healthcare providers, policy makers) to solicit wider input into the module selection process and to identify patient partner and academic co-leads to support the development of the individual modules. Based on these steps, an additional four modules were selected for development, making for a total of six modules:

1. A primer to diversity and patient engagement
2. LGBTQ2S+ communities
3. d/Deaf communities
4. Disabled communities
5. Rural and remote communities
6. Immigrant, racialized, refugee, and ethnocultural (IRRE) communities

### Module creation

Blended teams of patient partners and academic researchers developed the modules. Patient partners were engaged at the collaboration level of the International Association for Public Participation (IAP2) Spectrum [29]. We aimed to structure module teams to include two patient partners from the community identified in the module, one academic researcher, and a research trainee. This structure was decided from discussion with team members and drawing from Moss et al.’s [30] recommendation to create formal roles for deeper engagement of patient partners. However, module team structures evolved over time as the project progressed, resulting in teams with varying structures. Preference for team members was given to those who identified as a member of the community addressed in the module.

The project took an equity-oriented approach, attempting to address potential structural and individual inequities between patient partners and academic researchers, to ensure modules were co-created with equal input from team members. Patient partners were compensated through a lump sum, informed in part by the BC SUPPORT Unit Patient Partner Appreciation Policy [31]. Academic researchers were compensated only if they were not in current faculty positions; when they were compensated, the academic researchers were compensated the same amount as patient partners. Recruitment of module team members was conducted through informal networking, the BC SUPPORT Unit Patient Council, regional centers, and the Patient Voices Network. The project postdoctoral fellow (IWC) facilitated recruitment, orientation, training, and identification of roles. The co-investigators, including one patient partner co-lead (patient author BP), and two academic co-leads (authors EEM and SJB) provided additional support. The development team provided technical support, including guiding the scripting, storyboarding, videography and animation processes.

**Fig. 1:**
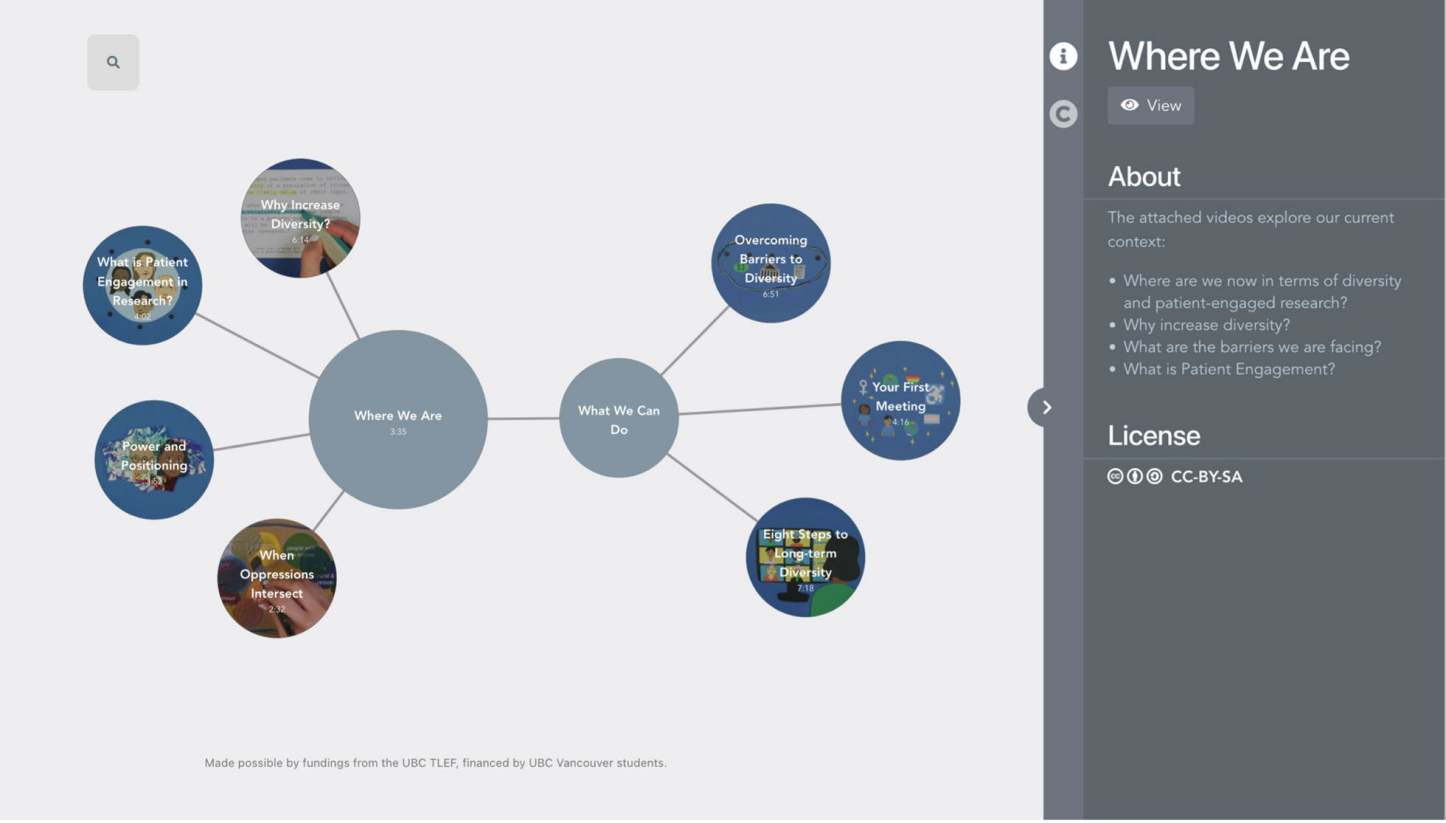
Example of the Tapestry Tool

The educational modules are now publicly available [32], and an example Tapestry is displayed in Fig. 1. Videos with an overview of module content can be viewed on the BC SUPPORT Unit YouTube channel [33].

### Module evaluation

#### Design

Evaluation of the modules was conducted using online surveys hosted on the Qualtrics platform. Participation involved giving informed consent, completing a pre-evaluation survey, viewing the module selected, and completing a post-evaluation survey. Participants were unable to add their own content for evaluation.

#### Participants

Participants were eligible for inclusion if they were health researchers, including trainees at or above the master’s level, over the age of 18, and resided in British Columbia.

Recruitment for evaluation occurred via informal networking, direct emailing of health researchers in British Columbia, asking organizations throughout BC to share study details among their networks, and social media posts (i.e., Twitter, LinkedIn, Facebook, and Instagram) by the BC SUPPORT Unit, team members, and partnering organizations. Throughout recruitment, organizations representing diverse communities were asked to share study details. A blog series was produced on the Collaborative RESearch Team to study psychosocial issues in Bipolar Disorder (CREST.BD) website, a research network led by EEM, that specializes in community-based participatory research (CBPR) in the mental health space and knowledge translation activities related to CBPR and POR.

#### Instruments

The pre-evaluation survey included three components: 1) demographic information, 2) module selected for evaluation, and 3) perceptions of diversity in POR. The post-evaluation survey included three components: 1) evaluation items on the content of the module, 2) the User Experience Scale Short Form (UES-SF, [34]), and 3) the same pre-evaluation items on participants’ perceptions of diversity in POR. All items were measured using five-point Likert scales from strongly disagree to strongly agree. Module content evaluation items included, for example, “I learned something new about engaging diverse people and communities in health research” and “the knowledge and tools in the online modules will allow me to better support diverse people and communities to engage in health research.” The UES-SF measures engagement on four subscales: focused attention (“feeling absorbed in the [modules] and losing track of time”), perceived usability (“experience as a result of [interacting with the modules] and the degree of control and effort expended”), aesthetic appeal (“the attractiveness and visual appeal of the interface”, and reward factor (“a valued experiential outcome”). The UES-SF includes evaluation items like “the online module was attractive” and “I felt engaged in this experience” [34]. In both the pre- and post-evaluation surveys, participants’ perceptions of diversity in POR were assessed using evaluation items modeled on the theory of planned behavior [35], which posits that three factors inform an individual’s intention to perform a specific behavior: attitudes, subjective norms, and perceived behavioral control [36]. Evaluation items for this section included measures of participants’ attitudes towards diversity in POR, perceptions of social norms of diversity in POR, and perceived behavioral control over engaging diverse people in POR [36]. Participants’ experiences towards diversity in POR were not assessed since this is not a factor considered in the theory of planned behaviour [35].

#### Data analysis

Prior to analysis, incomplete responses were discarded, and participants’ pre- and post-evaluation responses linked. Relevant variables were reverse coded so that in all cases 1 indicated a more negative experience and 5 indicated a more positive experience. For individuals who completed the pre-evaluation survey several times but the post-evaluation only once, only the most recent response was included in the analysis.

Descriptive statistics were used to summarize responses to items only included in the post-evaluation survey (i.e., module content and the UES-SF). Due to the skewed, non-normal distribution of the data, we conducted a sign test to assess changes in perceptions of diversity in POR pre- and post-evaluation. The median and range for each question is reported [37]. Due to the limited sample size in our study, we did not conduct comparative analyses between groups of participants (i.e. by module topic). All analyses were conducted in SPSS.

## Results

### Sample

The pre-evaluation survey received a total of 314 visits (i.e., the link to the pre-evaluation survey was opened 314 times). Of those, 78 (24.8%) individuals completed the pre-engagement survey but not the post-engagement survey. Due to the Tapestry Tool modules being housed on an external website to Qualtrics, we cannot determine the total number of people who viewed the module they had selected. Seventy-four participants completed study participation. One participant completed participation for two different modules; their second module evaluation was excluded from analysis. The primer module was selected most often for evaluation (31.1%, n = 23), and the d/Deaf module was least frequently selected (8.1%, n = 6). The LGBTQ2S+ and rural and remote modules were evaluated by 9 participants each (12.2%), the IRRE module was evaluated by 11 participants (14.9%), and the disabled module was evaluated by 16 participants (21.6%).

### Participant demographics

Table 1 displays participant demographics. Most participants identified as white (n = 58, 78.4%), women (n = 58, 78.4%), and working in affiliation with an academic institution (n = 42, 56.8%). The mean age of the sample was 40 years old (SD = 12.02), with a range of 23–74 years of age. Most participants were located in Vancouver (n = 53, 71.6%). Almost half of the participants identified as a member of LGBTQ2S+ , rural-remote, d/Deaf, and/or disabled communities (n = 43, 58.1%). Twenty-five participants (n = 25, 33.8%) evaluated a module corresponding to a community they identified with.

**Table 1:** Participant demographics

Variable	Total sample (*n* = 74)
Mean age	39.6 (SD = 12.02)
*Gender*	
Woman	58 (78.4%)
Man	13 (17.6%)
Non-binary/gender non-conforming	3 (4.1%)
*Race/ethnicity*	
White	58 (78.4%)
East Asian	10 (13.5%)
Latin American	2 (2.7%)
South Asian	1 (1.4%)
Middle Eastern	1 (1.4%)
Indigenous (Inuit, Métis, and/or First Nations)	0 (0.0%)
Black	0 (0.0%)
Decline to answer	2 (2.7%)
*Researcher status*	
Affiliated with an academic institution	42 (56.8%)
Trainee	21 (28.4%)
Clinician researcher	5 (6.8%)
Independent researcher	2 (2.7%)
Other	4 (5.4%)
*Region of residence in BC*	
Vancouver	53 (71.6%)
Fraser (includes Simon Fraser University downtown)	9 (12.2%)
Vancouver Island	7 (9.5%)
Northern	3 (4.1%)
Interior	2 (2.7%)
*Community identity*	
Immigrant, refugee, racialized and/or ethnocultural	19 (25.7%)
Disabled	13 (17.6%)
LGBTQ2S+	7 (9.5%)
Rural-remote	3 (4.1%)
d/Deaf	1 (1.4%)
None of the above	31 (41.9%)
*Member of the same community in module*	
Immigrant, refugee, racialized and/or ethnocultural	9 (12.2%)
Disabled	6 (8.1%)
LGBTQ2S+	5 (6.8%)
d/Deaf	3 (4.1%)
Rural-remote	2 (2.7%)
No	49 (66.2%)

### Module content

**Fig. 2:**
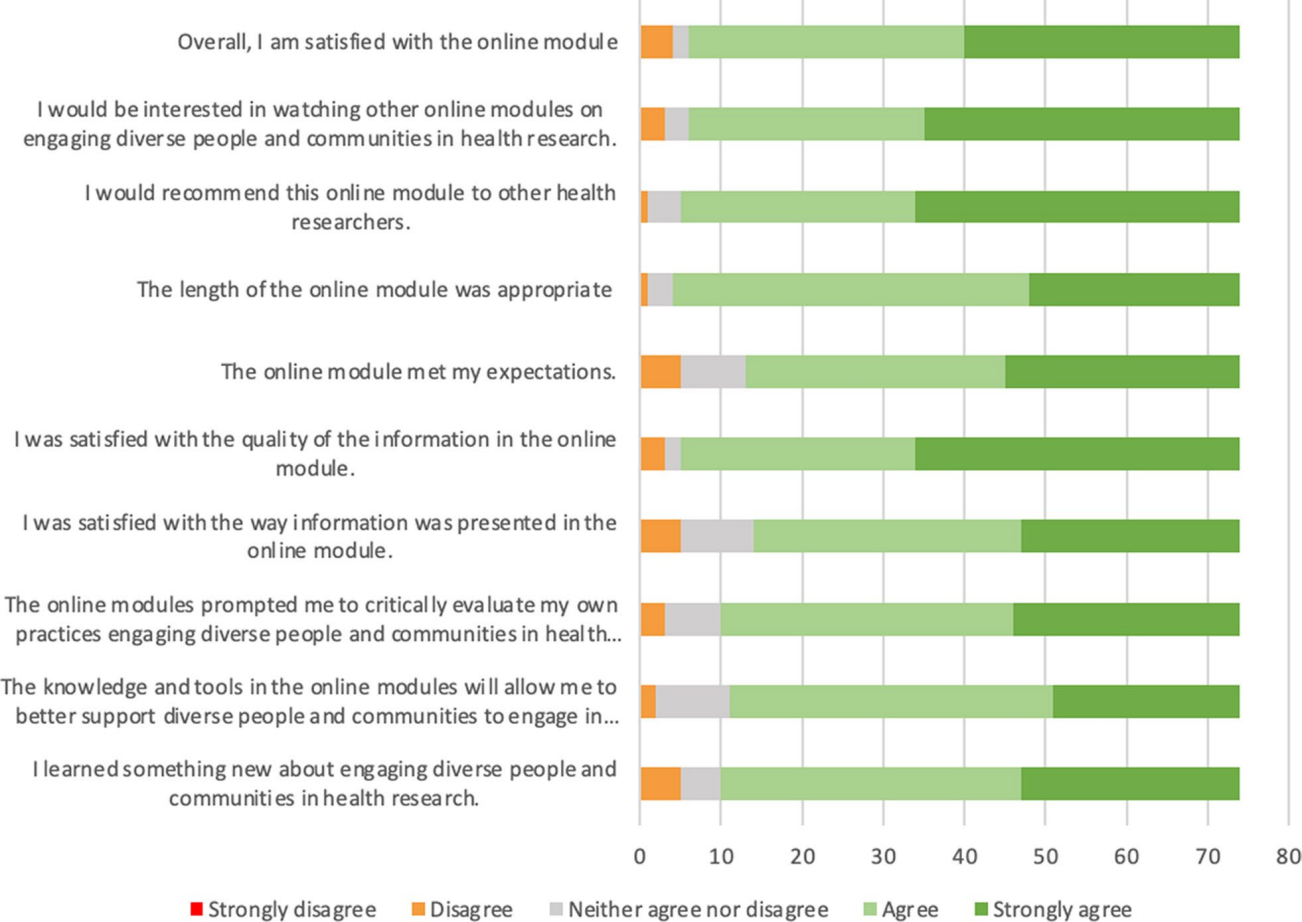
Summary of participants' responses to module content evaluation items

Evaluations of module content and presentation were generally positive (Fig. 2), with no participants indicating that they strongly disagreed with any evaluation items. More than 80% of participants agreed or strongly agreed with all of the module content evaluation items. Over 90% of the participants agreed or strongly agreed with the following evaluation items: “Overall, I am satisfied with the online module” (n = 68, 92.0%), “I would be interested in watching other online modules” (n = 68, 92%), “I would recommend this online module to other health researchers” (n = 69, 93.2%), and “I was satisfied with the quality of the information in the online module” (n = 69, 93.2%). The lowest proportion of responses of agree or strongly agree were for the following evaluation items: “I was satisfied with the way information was presented in the online module” (n = 60, 81.1%) and “The online module met my expectations” (n = 61, 82.4%). The evaluation items with the highest rate of neutral responses (i.e., “Neither agree nor disagree”) were the following: “The knowledge and tools in the online modules will allow me to better support diverse people and communities to engage in health research” (n = 9, 12.2%), “I was satisfied with the way information was presented in the online module” (n = 9, 12.2%), and “The online module met my expectations” (n = 8, 10.8%).

### The User Engagement Scale short form (UES-SF)

Results from the UES-SF are summarized in Table 2. Scores for the reward factor (4.21 ± 0.523) and aesthetic appeal (4.10 ± 0.715) were highest, with answers corresponding to agree or strongly agree. The mean score for the perceived usability was 3.93 ± 0.775, corresponding to agree. Scores for the focused attention scale were lowest, with the average corresponding to neither agree nor disagree (3.15 ± 0.753). The average total score of the UES-SF was 3.85 ± 0.530, corresponding to between agree and strongly agree.

**Table 2:** Mean and standard deviation of the user engagement scale short form

	Mean	Standard deviation
Focused attention	3.15	0.753
Perceived usability	3.93	0.775
Aesthetic appeal	4.10	0.715
Reward	4.21	0.523
Total score	3.85	0.530

NB: Items are scored on a Likert Scale, with 1 = Strongly Disagree and 5 = Strongly Agree. Higher scores indicate increased engagement.

### Perceptions of diversity in patient-oriented research

The mode and range of responses regarding participants’ perceptions of diversity in POR are summarized in Table 3. In the pre-evaluation, participants’ attitudes towards diversity in POR were positive, with a mode of agree or strongly agree. Participants’ perceived behavior control over diversity in POR was neutral: the mode response to evaluation items about having the necessary knowledge, confidence, and time/resources was neither agree nor disagree. Participants’ perceptions of norms relating to diversity in POR were positive (i.e., whether health researchers are expected to use specific methods to engage diverse people in POR), with all evaluation items having a mode response of agree. Changes in participant responses, as analyzed by the sign test, are summarized in Table 4. A positive change indicates a participant answered higher on the Likert scale on the post-evaluation survey than the pre-evaluation survey (e.g., scored a 3 on pre-evaluation survey and a 4 on post-evaluation survey), and vice versa for negative change. The sign test indicated a statistically significant change in response to six of the ten evaluation items, all of which were positive changes.

**Table 3:** Mode and range of participants' beliefs about diversity in POR at pre- and post-evaluation

	Pre-evaluation			Post-evaluation		
	Mode	Range		Mode	Range	
		Min	Max		Min	Max
*Attitudes towards diversity in POR*						
Engaging diverse people and communities in health research will result in higher quality research	5.00	4.00	5.00	5.00	3.00	5.00
The experience of engaging in health research is beneficial for diverse people and communities	4.00	2.00	5.00	5.00	3.00	5.00
Specific methods are required to engage diverse people and communities in health research	5.00	3.00	5.00	5.00	3.00	5.00
*Perceived behavioral control over diversity in POR*						
I have the necessary knowledge on specific methods to engage diverse people and communities in health research	3.00	1.00	5.00	4.00	3.00	5.00
I am confident in my ability to implement specific methods to engage diverse people and communities in health research	3.00	1.00	5.00	4.00	1.00	5.00
I have adequate time and resources to implement specific methods to engage diverse people and communities in health research	3.00	1.00	4.00	3.00	2.00	5.00
*Subjective norms towards diversity in POR*						
Patients and the public value the inclusion of diverse people in health research	4.00	2.00	5.00	4.00	2.00	5.00
People who I work with think that I should be implementing specific methods to engage diverse people and communities in health research	4.00	2.00	5.00	4.00	2.00	5.00
It is expected that health researchers in Canada will use specific methods to engage diverse people and communities in health research	4.00	2.00	5.00	4.00	2.00	5.00
I intend to apply specific methods to engage diverse people and communities in health research	4.00	3.00	5.00	4.00	3.00	5.00

NB: Items are scored on a Likert Scale, with 1 = Strongly Disagree and 5 = Strongly Agree

**Table 4:** Results of sign test: comparing participants beliefs about diversity in POR at pre- and post-evaluation

Item	Positive change n (%)	Negative change n (%)	No change n (%)	Z-Score
*Attitudes towards diversity in POR*				
Engaging diverse people and communities in health research will result in higher quality research	2 (2.7)	7 (9.7)	65 (87.8)	
The experience of engaging in health research is beneficial for diverse people and communities	18* (24.3)	6 (81.1)	50 (67.6)	
Specific methods are required to engage diverse people and communities in health research	15 (20.3)	12 (16.2)	47 (63.5)	− 0.385
*Perceived behavioral control over diversity in POR*				
I have the necessary knowledge on specific methods to engage diverse people and communities in health research	37*** (50.0)	4 (5.4)	33 (44.6)	− 4.998
I am confident in my ability to implement specific methods to engage diverse people and communities in health research	33*** (44.6)	3 (4.1)	38 (51.4)	− 4.833
I have adequate time and resources to implement specific methods to engage diverse people and communities in health research	22** (29.7)	6 (8.1)	46 (62.2)	− 2.835
*Subjective norms towards diversity in POR*				
Patients and the public value the inclusion of diverse people in health research	11 (14.9)	11 (14.9)	52 (70.3)	
People who I work with think that I should be implementing specific methods to engage diverse people and communities in health research	20* (27.0)	8 (10.8)	46 (62.2)	− 2.079
It is expected that health researchers in Canada will use specific methods to engage diverse people and communities in health research	20* (27.0)	6 (8.1)	48 (64.9)	− 2.550
I intend to apply specific methods to engage diverse people and communities in health research	13 (17.6)	12 (16.2)	49 (66.2)	

NB: *p < .05, **p < .01, ***p < .001. Z scores are not available for items with a low sample size (i.e., low numbers of positive or negative change)

One out of the three evaluation items relating to participants’ attitudes towards diversity in POR showed a statistically significant increase in the positivity of responses: “The experience of engaging in health research is beneficial for diverse people and communities.” 18 participants responded more positively in the post-evaluation compared with the pre-evaluation survey, and the mode changed from agree to strongly agree (*p* = .023).

All three evaluation items relating to perceived behavioral control over diversity in POR showed significant positive changes. The question with the highest number of positive changes between pre- and post-evaluation was “I have the necessary knowledge on specific methods to engage diverse people and communities in health research,” with 37 participants responding more positively in the post-evaluation compared with the pre-evaluation survey, and the mode changing from neither agree nor disagree to agree (z = − 4.998, *p* < .001). Thirty-three participants responded more positively to “I am confident in my ability to implement specific methods to engage diverse people and communities in health research” (z = − 4.833, *p* < .001), and the mode increasing from neither agree nor disagree at pre-evaluation to agree at post-evaluation. Twenty-two participants indicated a more positive response to “I have adequate time and resources to implement specific methods to engage diverse people and communities in health research” (z = − 2.835, *p* = .005), with the mode being neither agree nor disagree at both time points.

Two out of the four evaluation items related to subjective norms towards diversity in POR showed a statistically significant positive change. Twenty participants indicated a more positive response to “People who I work with think that I should be implementing specific methods to engage diverse people and communities in health research” (z = − 2.079, *p* = .038) and “It is expected that health researchers in Canada will use specific methods to engage diverse people and communities in health research” (z = − 2.550, *p* = .011), with the mode for both evaluation items being agree at both pre- and post-evaluation time points.

## Discussion

POR in healthcare settings is often limited to engaging with patients from similar backgrounds and lived experiences: white, women, and middle class [11]. Furthermore, a lack of diversity within research teams means that patients and researchers are more likely to belong to similar social and educational backgrounds, thus encouraging a preponderance of shared values and decision-making relevant mostly to the dominant culture. A lack of inclusion of certain populations in authentic patient engagement runs the risk of developing and evaluating healthcare services on the basis of the concerns and priorities of the dominant population, which may “further entrench health inequities and preclude the ability to surface ideas that challenge dominant conceptualizations of health and healthcare, thereby reinforcing the status quo rather than promoting healthcare transformation” [38]. Despite a recognized need for specific methods to engage with diverse people in health research, research is still growing about best practices for health researchers to effectively engage with diverse communities. This study aimed to address the gap in knowledge of POR methods through the co-creation and preliminary evaluation of a suite of online educational modules for health researchers. Results from the preliminary evaluation indicate the modules were engaging. After viewing the modules, participants were significantly more likely to agree that they have the necessary knowledge, ability, time, and resources to engage diverse people in POR. In addition, participants were more likely to agree that engaging in health research is beneficial to diverse communities and that the people they work with expect them to engage diverse communities.

Results from the UES-SF suggest that the Tapestry Tool is an engaging platform to deliver education to health researchers. In particular, the aesthetic appeal and reward factor of the modules was high, with first-time users finding the tool easy to navigate and use. High ratings for the reward factor of the modules may have been related to the high ratings for module content; participants indicated they learned something new and that the content of the modules will allow them to improve their research practices.

Participant responses to evaluation items about the quality and presentation of module content were also high. In part this may reflect the way in which the modules were co-created. Lam and Shulha [39], in their co-creation of an educational program, indicate that co-creation of educational content may leverage both group’s expertise, thereby creating a more useful, and relevant educational resource. Furthermore, in healthcare quality improvement settings, co-creation may improve the applicability of research learnings and efficiency of research more generally [40]. In our study, input from both academic researchers and patient partners may have helped ensure that the content was directly relevant to health researchers, while still prioritizing critical issues from patient perspectives.

While educational modules provide knowledge and tools, this knowledge needs to affect behavior to increase diversity in health research. The theory of planned behavior [35] posits that three factors influence behavior: personal attitudes, perceived subjective norms (i.e., perceptions of how others or society at large views a behavior), and perceived behavioral control. The modules significantly impacted elements of these three pillars. Participants’ perceived behavioral control over diversity in POR showed the strongest significant difference between pre- and post-evaluation. This finding is reflected in the content evaluation items: most participants agreed the online modules prompted critical self-reflection and will allow them to better engage with diverse communities. A lack of knowledge surrounding best practices for effective engagement is one barrier to diversity in POR [6, 12, 14]. Increasing health researchers’ perceived behavioural control over diversity in POR, through the provision of tools and knowledge to do so, may help to bridge the gap between the importance of diversity in POR and pragmatic challenges that prevent effective engagement with diverse people. It may not be surprising that changes to participants’ perceived behavioral control were strongest: the mode for evaluation items related to attitudes and subjective norms towards diversity in POR in pre-evaluation were high, all corresponding to agree or strongly agree. Given that the sample already viewed diversity in POR as beneficial and as a societal expectation, agreement with these evaluation items was less likely to be increased by the modules.

The co-creation of the modules with patient partners was fundamental to this project; the content of the educational modules is much richer and more comprehensive than it would have been without lived experience input. Patient partners were involved not only in content creation but also in recruiting participants for evaluation and in sharing the final modules. We are pleased to have been able to navigate working with six different teams to create modules that both the academic and patient partner collaborators are proud of. As a result, many team members are invested in promoting the modules for a wider reach. Additionally, all the patient partners involved in this project are members of *hardly*-reached communities, and many were engaging in POR for the first time. It is our hope that this project assisted with capacity building and has left a legacy for increased diversity in future POR projects. However, this successful outcome did not come without challenges. The COVID-19 pandemic played a significant role in each of these, including the teams having to navigate relationship building using new technology, a shift in group dynamics from not being able to meet in person, and high personnel turnover. The high turnover in module teams can be attributed to several factors: changes in responsibilities due to the pandemic, researcher and patient partners leaving due to health issues, and structural power imbalances negatively impacting relationships within the teams. The delayed payment of patient partners was one major structural barrier to inclusion: invoices sometimes took months to process and required repeated (often unsuccessful) follow-up with the institution’s finance department. This problem is an indication of a research system that has not yet adapted to the requirements and realities of effective patient engagement. Similarly, working with research partners who may not be able to attend scheduled meetings or meet project deadlines because of their physical or mental health, or other competing obligations, involves flexibility. However, existing research and funding schedules do not accommodate, highlighting that the research ecosystem must continue to evolve to meet the requirements of authentic patient engagement [20]. Finally, although we aimed to be trauma and resiliency informed, upon reflection, the recruitment process itself should have included more trauma and resiliency informed approaches [41], such as asking patient partners if they wanted to divulge specific trauma activators and planning the project and meetings with consideration of those triggers.

Each of the modules highlights the importance of building trust within communities to help diversify patient partners involved in research, requiring long-term commitment. A study interviewing health researchers about researcher-level barriers to diversity in translation research highlights the need for planning events within the community to build trust and rapport with *hardly*-reached communities, which takes time and investment [20]. Perceived barriers for health researchers to addressing lack of diversity in health research include time, resource, and cost considerations [3–7, 20]. Heller et al. [42], in their systematic review of addressing barriers to clinical trial enrolment for racialized communities, argue that a longitudinal approach to diversity in health research may reduce recruitment costs for some projects. In addition, each of the modules evaluated in this project highlighted the need for diverse or tailored recruitment strategies for different communities: while concepts between modules may overlap, strategies from each module can be applied to support engagement across and within specific communities of interest. Finally, researchers aiming to apply the concepts from these modules may consider the importance of intersectionality: patient partners may belong to one, none, many, or all the communities covered in the modules. Careful consideration of intersecting identities is integral to ensuring representative patient partners: as discussed in the disabled communities module, disability representation “has a whiteness problem.” Recruiting solely from large disability organizations may perpetuate this lack of diversity. However, recruiting from disability organizations specifically for Black and/or racialized community members, in conjunction with broader organizations, may be appropriate. Our modules represent a starting point for health researchers aiming to engage with more diverse patient partners: careful consideration and tailoring to the community in which the research is conducted is required to begin to address barriers to inclusion.

Our results indicate the modules were engaging and may shift attitudes towards diversity in POR, thereby presenting the co-creation process and Tapestry Tool platform as viable options for increasing engagement with diverse communities in health research. However, there are several notable limitations to our study. Firstly, the small number of participants, homogenous nature of our sample, and skewed nature of the data limited statistical analysis. Future studies may look to assess the demographics of health researchers who respond best to the modules (i.e., age, field of study, experience level) to further gauge how to tailor modules for specific settings. Furthermore, it is likely that the health researchers who evaluated the modules already have an interest in increasing diversity and therefore are amenable to adjusting their practices to do so. However, research into how best to reach researchers with the least diversity in their patient engagement, who might benefit from this guidance the most, would help inform dissemination and maximize the impact of educational modules. Secondly, although rooted in the theory of planned behavior, survey evaluation items are a proxy for actual behavior change. Further work is required to assess whether educational offerings such as these modules impact real-world behaviors. Third, modules were longer than intended due to the amount of material that needed to be included: each module was between 30 and 40 min, which may have deterred participation. Finally, although these modules represent a wide range of community members, this project is in its initial stages, and as such, there are many important communities not represented here. People may belong to none, one, or many of these groups, requiring thoughtful reflection on how the intersectional identities of patient partners need to be considered when engaging with communities. In addition, as diverse as our patient partners were, they still represented only a limited perspective. Historically marginalized groups are not monolithic, and there may be considerations—some of which may conflict with what we have presented—that the modules are missing. The fact that they are now published in a flexible format may help the modules capture missing perspectives in the future.

This study indicates online modules may be one way to support health researchers in engaging with more diverse patients. Increasing researchers’ awareness and understanding of systemic barriers to patient engagement in health research and providing health researchers with tools to begin to address these barriers, is an initial step to increasing diversity [10]. However, individual behavior change must occur in tandem with broader-scale changes to health research practices to address systemic structural inequalities, such as disenfranchisement, racism, oppression, and stigma and discrimination [10].

## Conclusion

Patient engagement research stands to benefit from hearing diverse patient perspectives; the current lack of diversity limits the generalizability and impact of research findings and the ability of research to address health disparities. Online educational modules may represent one way of empowering health researchers to engage with more diverse patient partners, by providing them with knowledge and tools for engagement best practices.

## Supplementary Information

**Additional file 1**: A short form reporting checklist to guide the reporting of patient and public involvement in health and social care research.

## Data Availability

The datasets used or analyzed during the current study are available from the corresponding author on reasonable request.

## Abbreviations

CIHR: Canadian Institutes of Health Research
POR: Patient-oriented research
SPOR: Strategy for Patient-Oriented Research
BC SUPPORT Unit: British Columbia SUpport for People & Patient-Oriented Rresearch & Trials Unit
CREST.BD: Collaborative RESearch Team to study psychosocial issues in Bipolar Disorder
IRRE: Immigrant, refugee, racialized and ethnocultural
LGBTQ2S+: Lesbian, gay, bisexual, transgender, queer/questioning, two-spirit. The + represents additional gender and sexually diverse groups.
UES-SF: User Engagement Scale short form
