# Supplementary material for: Engaging diverse patients in a diverse world: the development and preliminary evaluation of educational modules to support diversity in patient engagement research
Source: Res Involv Engagem. 2023 Jul 7;9:47. doi: 10.1186/s40900-023-00455-0 (PMC10327362; doi:10.1186/s40900-023-00455-0)
Supplement: Supplementary file 1 — Additional file 1: A short form reporting checklist to guide the reporting of patient and public involvement in health and social care research. [file 40900_2023_455_MOESM1_ESM.pdf]

Section and topic	Item	Reported on page No
1: Aim	Report the aim of PPI in the study	4
2: Methods	Provide a clear description of the methods used for PPI in the study	4-5
3: Study results	Outcomes—Report the results of PPI in the study, including both positive and negative outcomes	N/A
4: Discussion and conclusions	Outcomes—Comment on the extent to which PPI influenced the study overall. Describe positive and negative effects	N/A
5: Reflections/critical perspective	Comment critically on the study, reflecting on the things that went well and those that did not, so others can learn from this experience	10-12

PPI patient and public involvement
